# Effects of Telbivudine Treatment on the Circulating CD4^+^ T-Cell Subpopulations in Chronic Hepatitis B Patients

**DOI:** 10.1155/2012/789859

**Published:** 2012-04-11

**Authors:** Yanhua Zheng, Zemin Huang, Xianhua Chen, Yi Tian, Jun Tang, Yi Zhang, Xiaomin Zhang, Jijun Zhou, Qing Mao, Bing Ni, Qinghong Wang, Yuzhang Wu

**Affiliations:** ^1^Institute of Immunology, Third Military Medical University, PLA, Chongqing 400038, China; ^2^Department of Pathology and Experimental Medicine, 306 Hospital, PLA, Beijing 100101, China; ^3^Department of Health Care, Southwest Hospital, Third Military Medical University, Chongqing 400038, China; ^4^Department of Dermatology, 105th Hospital, PLA, Hefei 230001, China; ^5^Department of Infectious Diseases, Southwest Hospital, Third Military Medical University, Chongqing 400038, China; ^6^Ministry of Education Key Laboratory of Child Development and Disorders, Pediatric Research Institute, Children's Hospital of Chongqing Medical University, Chongqing 400014, China

## Abstract

CD4^+^ T cells serve as master regulators of the adaptive immune response to HBV. However, CD4^+^ T-cell subsets are heterogeneous, and it remains unknown how the antiviral agents affect the different CD4^+^ T cell subtypes. To this end, the expressions of signature transcription factors and cytokines of CD4^+^ T-cell subtypes were examined in hepatitis B patients before and after treatment with telbivudine. Results showed that, upon the rapid HBV copy decrease induced by telbivudine treatment, the frequencies and related cytokines of Th17 and Treg cells were dramatically decreased, while those for Th2 cells were dramatically increased. No obvious changes were observed in Th1 cell frequencies; although, IFN-**γ** expression was upregulated in response to telbivudine treatment, suggesting another cell source of IFN-**γ** in CHB patients. Statistical analyses indicated that Th17 and Tr1 (a Treg subtype) cells were the most sensitive subpopulations of the peripheral blood CD4^+^ T cells to telbivudine treatment over 52 weeks. Thus, Th17 and Tr1 cells may represent a suitable and effective predictor of responsiveness during telbivudine therapy. These findings not only improve our understanding of hepatitis pathogenesis but also can aid in future development of appropriate therapeutic strategies to control viral hepatitis.

## 1. Introduction

Persistent infection with the hepatitis B virus (HBV) remains a challenging global health problem. Currently more than 370 million people are chronically infected, and this rate is estimated to increase by 4 million per year. Hepatitis B is a leading cause of chronic hepatitis, cirrhosis and hepatocellular carcinoma and accounts for about 1 million deaths annually [1].

The pathogenesis of liver damage during an HBV infection is immune-mediated and is dependent on the balance between viral replication and the host HBV-specific T-cell response [2, 3]. In patients with an acute self-limiting HBV infection, a multispecific T-cell response is important for control of the infection [4, 5]. Patients with a chronic HBV infection, however, lack such a vigorous multispecific T-cell response and instead exhibit a weak or undetectable virus-specific T-cell response [4]. This condition of T cell hyporesponsiveness is frequently associated with high viral and/or antigen load in chronic hepatitis B (CHB) patients [6, 7]. Therefore, early inhibition and decrease of HBV replication is likely essential for the recovery of T-cell reactivity in CHB patients.

Several currently available drugs are approved for treatment of HBV infection and in routine use, including interferon-*α* (IFN-*α*), pegylated IFN*α*-2a, lamivudine, adefovir, and entecavir. The newly developed nucleoside analogue, telbivudine, has also been approved for clinical use by both the US Food and Drug Administration and the European Medicines Evaluation Agency [8]. It has potent and specific anti-HBV activity, which is triggered upon phosphorylation by cellular kinases. The active triphosphate form of telbivudine can then inhibit the HBV DNA polymerase activity by competing with its natural substrate thymidine 5′-triphosphate [9]. In this manner, telbivudine can not only effectively suppress HBV replication and propagation but also decrease the consequent liver injury [10–12]. Moreover, these drug effects are also accompanied by marked increases in CD4^+^ and CD8^+^ T cell responsiveness [13–17]. Chen et al. demonstrated that telbivudine treatment can lead to an increased frequency of peripheral blood CD4^+^T lymphocytes and an augmented proliferative response of HBV-specific T cells to the hepatitis B core antigen (HBcAg) [18].

CD8^+^ T cells are considered the key cellular effectors mediating HBV clearance from the liver, and they produce this effect through a Fas-dependent and perforin-independent process [19]. However, induction of the CD8^+^ T cell response is dependent upon the presence of CD4^+^ T cells, as evidenced by an HBV model in which CD4^+^ T cells were found to serve as the master regulators of the adaptive immune response [19]. Boni et al. also demonstrated that CD8^+^ T cell responses were enhanced markedly in HBV infection and showed that this enhancement followed the reconstitution of CD4^+^ reactivity and the decline of viral load induced by lamivudine therapy [13, 15]. Collectively, these data indicated a pivotal role for CD4^+^ T cells in anti-HBV immunity. However, CD4^+^ T cells are a heterogeneous population, comprised of at least four subtypes (Th1, Th2, Treg, and Th17 cells), each with distinctive functions. Dissecting the precise roles of each CD4^+^ T cell subtype will provide insights into the host mechanisms underlying the pathogenesis and persistence of HBV infection.

To gain a more detailed understanding of the roles played by each of the circulating CD4^+^ T-cell subpopulations in chronic HBV infection, we performed a longitudinal investigation of the changes in CD4^+^ T-cell subpopulations following rapid decrease of viral load induced by telbivudine treatment. Furthermore, we determined which of the main subtypes were closely correlated with the efficacy of such treatment. The findings from our study are expected to open new avenues for treatment of chronic hepatitis B based on induction of the beneficial CD4^+^ T cell subtypes by combining antiviral agents and specific T-cell stimulators or suppressors.

## 2. Methods and Materials

### 2.1. Patients

Thirty-eight chronic hepatitis B patients (24 males and 14 females, aged 21 to 50 years; Table 1), negative for markers of human immunodeficiency virus, hepatitis C, and hepatitis D infection, were enrolled from the Hepatitis Clinic of Southwest Hospital (Chongqing, China). Heparinized blood samples were collected from each patient at baseline (0) and at 12, 24, 36, and 52 weeks after telbivudine treatment (600 mg orally per day). Control blood samples were donated by 23 healthy individuals (15 males and 8 females, aged 25 to 45 years). All patients were positive for HBV e antigen (HBeAg) at baseline and had biopsy-proven chronic hepatitis with various degrees of liver inflammation and fibrosis. Written informed consent was obtained from each patient prior to study participation, and the study protocol was approved by the Ethics Committee of Southwest Hospital.

### 2.2. Cell Isolation

Peripheral blood mononuclear cells (PBMCs) were isolated from fresh heparinized peripheral blood by using the Optiprep Nycoprep Lymphoprep kit (Axis-shield, Oslo, Norway) according to the manufacturer's protocol. Isolated cells were suspended in Trizol reagent (Invitrogen, Karlsruhe, Germany) and frozen in −80°C until use for mRNA extraction. The plasma from the same peripheral blood sample was collected and frozen in −80°C until use in the enzyme-linked immunosorbent assay (ELISA).

### 2.3. Cytokines

Cytokines in serum (after removal of fibrinogen from plasma) were detected by conventional sandwich ELISA using detection and capture Abs from eBioscience (IL-17A and IL-23; San Diego, CA, USA) and Dakewei (IL-4, IL-10, TGF-*β*1, and IFN-*γ*; Beijing, China). Extinction spectra and cytokines were measured at 450 nm on an ABS-MONO spectrophotometer (s/n 1105; Beckman, Krefeld, Germany) and with the accompanying Multimode Analysis software. ELISAs were carried out following the respective manufacturer instructions. Standards and samples were run in duplicate.

### 2.4. Reverse Transcriptase Polymerase Chain Reaction (RT-PCR) and Quantitative (q)PCR

Extraction of total RNA was performed using the Trizol reagent (Invitrogen) and following the manufacturer instructions. The isolated RNA (2.5 *μ*g) was applied as template to a reverse transcription reaction using the One Step SYBR PrimeScript RT-PCR kit (Perfect Real Time; TaKaRa, Shiga, Japan). The qPCR reaction was performed with an Mx3000P qPCR system (Agilent Technologies, Waldbronn, Germany) using the SYBR Premix *Ex Taq* (Perfect Real Time; TaKaRa) and following the manufacturer guidelines. The following gene-specific primer pairs were used: FOXP3: (forward) 5′-AAG GAA AGG AGG ATG GAC G-3′ and (reverse) 5′-CAG GCA AGA CAG TGG AAA CC-3′; T-bet: (forward) 5′-ATG TGA CCC AGA TGA TTG TG-3′ and (reverse) 5′-CGG AAA GTA AAG ATA TGC GTG-3′; ROR*γ*t, (forward) 5′-ACT CAA AGC AGG AGC AAT GGA A-3′ and (reverse) 5′-AGT GGG AGA AGT CAA AGA TGG A-3′; GATA3, (forward) 5′-TAG CTG TAA GGC ATG AAG GAT G-3′ and (reverse) 5′-CTG GTG AAC GGT AAC ACT GAT T-3′; GAPDH, (forward) 5′-CGG AGT CAA CGG ATT TGG TCG TAT-3′ and (reverse) 5′-AGC CTT CTC CAT GGT GGT GAA GAC-3′. For each primer pair, a standard curve was generated using different cDNA dilutions to calculate mRNA expression of the respective gene. Next, each mRNA expression level was normalized to the GAPDH expression in the respective cDNA preparation. Controls were arbitrarily set to 1.0, and gene induction was calculated as fold increase compared to the controls.

### 2.5. Statistical Analysis

Statistical analysis was performed with the SPSS software program v14.0 (Chicago, IL, USA). All results at different time points are presented as means ± standard error of the mean (SEM) or as median with range. Differences among groups were compared using the Student's *t*-test and Mann-Whitney *U* test. Correlations were evaluated using Spearman's rank correlation test for nonparametric values. Differences were considered statistically significant at *P* < 0.05.

## 3. Results

### 3.1. Serological and Virological Response to Telbivudine Therapy in CHB Patients

**Figure 1:**
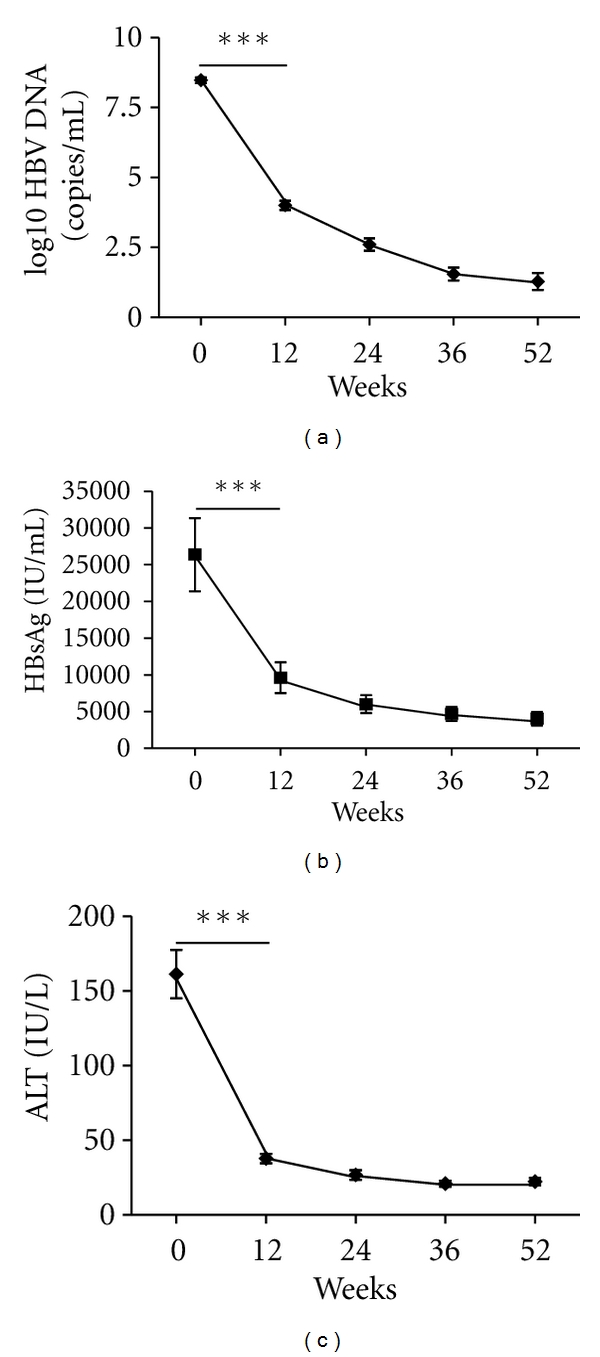
Clinical characteristics of CHB patients after treatment with telbivudine. Serum samples were collected from 38 CHB patients treated with telbivudine at five time points after treatment initiation. HBV DNA level was detected by qPCR. Serum HBsAg concentration was detected by ELISA. Serum ALT was measured by the CHEMIX-180 automated biochemistry analyzer (Japanese SYSMEX Corporation). ***P < 0.001 versus  baseline (0).

During the entire 52-week therapy course, the viremia of all 38 patients showed an immediate sharp decline to a low or undetectable level, which was maintained until the end of the therapy (Figure 1). In addition, eight patients were found to have seroconverted to the hepatitis B e antibody (anti-HBe) after 36 weeks of treatment; the remaining 30 patients stayed HBeAg-positive throughout the 52 weeks of treatment. In accordance with the sharp decrease observed in HBV copy number in serum, the HBsAg concentration was found to be remarkably reduced after 12 weeks of treatment, and this low level remained stable through the remainder of the treatment course (Figure 1). Following the rapid virus clearance after treatment, liver injury was greatly alleviated, as evidenced by remarkably reduced serum ALT levels starting at 12 weeks of treatment (Figure 1).

### 3.2. Telbivudine Decreases the Population of Treg and Th17 Cells but Increases the Amount of Th2 Cells

**Figure 2:**
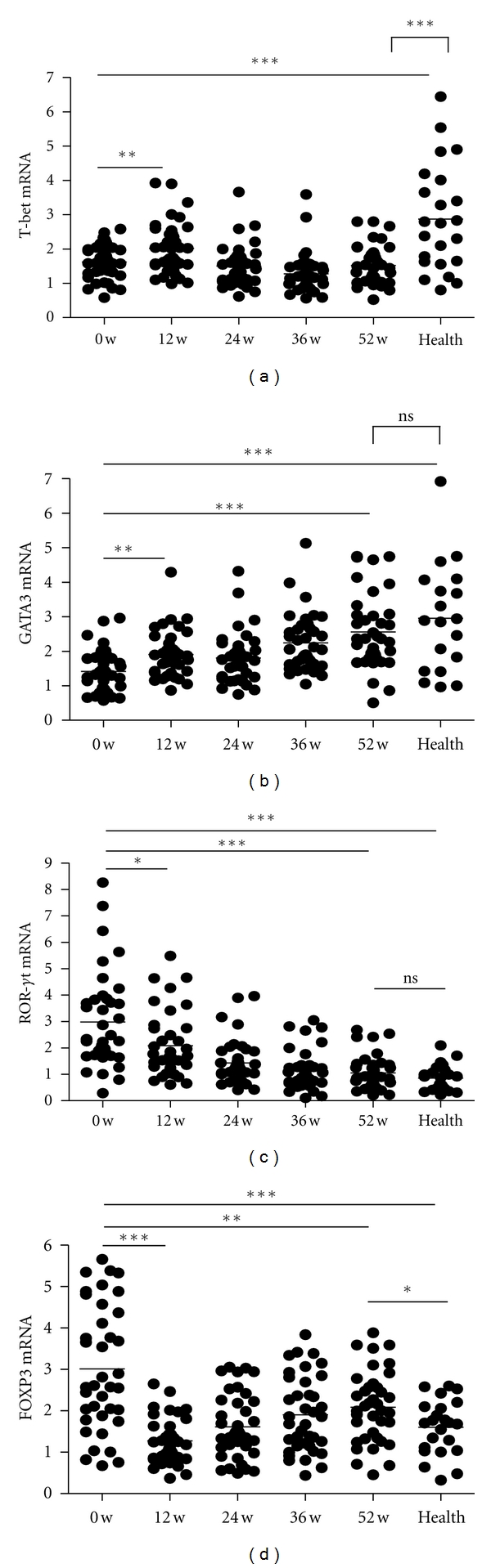
Changes in mRNA expressions of critical transcriptional factors for CD4^+^ T-cell subpopulations in response to telbivudine treatment. PBMCs from the healthy controls and CHB patients treated with telbivudine were used to detect the relative mRNA expression levels of the CD4^+^ T-cell subtype-specific transcription factors by qPCR. *P < 0.05, **P < 0.01, and ***P < 0.001  versus the respective baseline or 52-week value. Ns: no significance.

Since the above results demonstrated excellent therapeutic effects of telbivudine on HBV propagation inhibition and liver injury alleviation, we investigated the mechanisms underlying such therapeutic effects. CD4^+^ T cells are known to play very important roles in antivirus immunity and to mediate liver injury. Thus, we investigated whether CD4^+^ T-cell subpopulations were influenced by telbivudine treatment by using qPCR to measure the expression levels of CD4^+^ T-cell subtype-specific transcription factors. Results showed that telbivudine had different effects on the different CD4^+^ T-cell subtypes (Figure 2). The mean expression level of T-bet, the critical transcription factor of Th1 cells, in PBMCs became obviously increased after 12 weeks of treatment but then declined and stabilized at the baseline level until the end of the treatment course (week 52). Furthermore, the T-bet expression levels in HBV-infected patients were markedly lower than those in healthy controls throughout the entire treatment course. Unlike the Th1-specific T-bet expression profile, the critical transcription factor of Th2 cells, GATA3, increased gradually in response to treatment, eventually reaching the level of the healthy controls after 52 weeks of treatment. For the Th17 cells, the expression of ROR*γ*t in PBMC was found to decrease steadily in response to telbivudine treatment, reaching the normal level at 52 weeks of treatment. Similarly, the mRNA expression of the Treg-specific FOXP3 gene was found to decrease over time in response to telbivudine treatment, until the level reached close to the normal level (*P* = 0.03).

### 3.3. Telbivudine Influences the Expression of the Key Cytokines in CD4^+^ T-Cell Subtypes Asynchronously

Each of the CD4^+^ T-cell subtypes differentiates from naïve CD4^+^ T-cells in certain permissive microenvironments and secretes the distinctive profiles of cytokines. Based on this knowledge, we detected the signature cytokines of each CD4^+^ T-cell subtype in the serum of patients by using ELISA. As shown in Table 2, telbivudine influenced the expression of all the key cytokines in CD4^+^ T-cell subtypes, except for IFN-*γ*, in a pattern very similar to that observed for the signature transcriptional factors (Figure 2). Telbivudine treatment caused a gradual decrease in IL-17 and IL-23 (Th17-related cytokines) and TGF-*β* and IL-10 (Treg cell-related cytokines) but increased IL-4 (Th2 cell cytokine). No significant change was observed in the T-bet expression levels after 52 weeks of treatment (Figure 2); however, there was a significant increase in the expression of Th1-related cytokine, IFN-*γ*, after 24 weeks of treatment (Table 2). All the detected cytokines restored to normal levels except IL-10 that was significantly decreased but still higher than healthy control at the end of treatment (Table 2).

### 3.4. Correlation between the CD4^+^ T-Cell Subtypes and HBV DNA Levels in Telbivudine-Treated CHB Patients

Telbivudine ameliorated the virological and serological response and altered the expressions of various CD4^+^ T-cell transcription factors and the related signature cytokines in CHB patients. We next examined whether the improved clinical phenotypes were caused by the altered CD4^+^ T-cell population in telbivudine-treated CHB patients by analyzing the correlation between the two.

**Figure 3:**
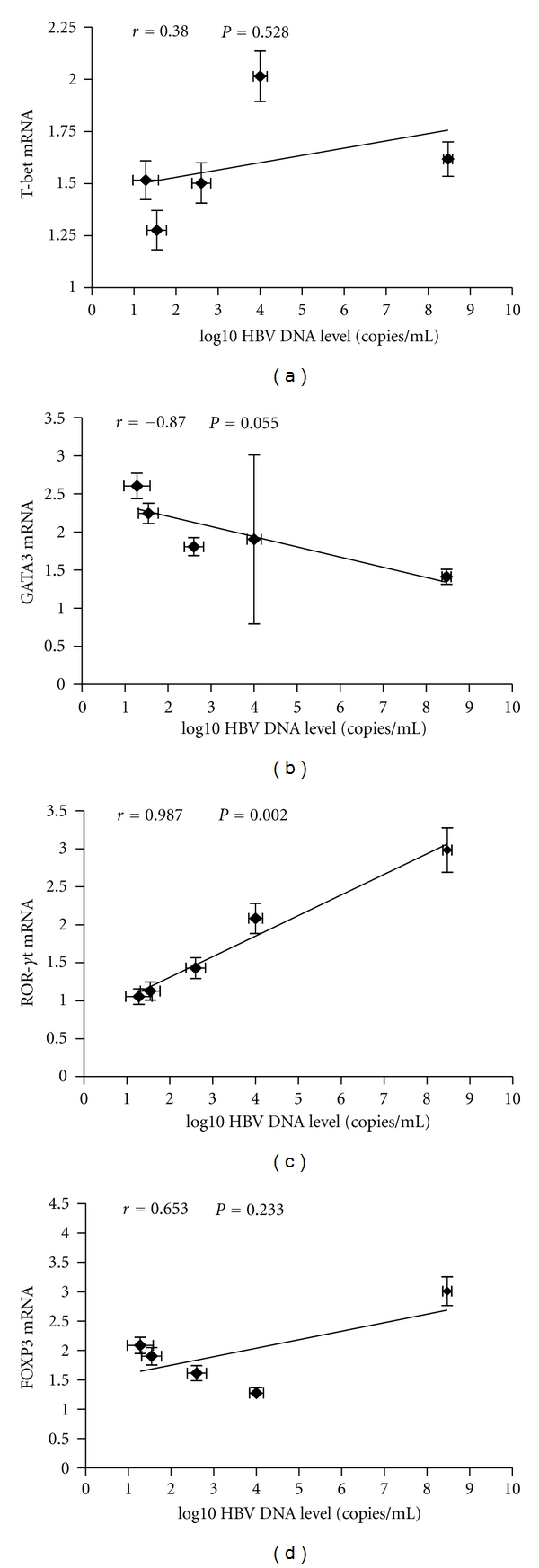
Correlation between the signature transcription factors of CD4^+^ T-cell subtypes and HBV DNA level. Expressions of the signature transcription factors for each of the CD4^+^ T-cell subtypes and HBV DNA level at various time points of treatment (baseline and weeks 12, 24, 36, and 52) are indicated on the plots by diamonds. Error bars indicate SEM.

Telbivudine is highly selective for HBV DNA and inhibits viral DNA synthesis with no effect on human DNA or other viruses. Our results clearly indicated that telbivudine suppressed the HBV propagation; however, following the decrease of HBV copies in serum, the frequency and function of the CD4^+^ T-cell subpopulations were also changed. Since the particular CD4^+^ T-cell subtype influenced by the decrease of HBV copies can reflect the mechanisms involved in HBV infection, we further analyzed the relationship between CD4^+^ T-cell subtypes and HBV DNA level. A significant correlation was found between the Th17-related transcription factor and HBV DNA levels in the telbivudine-treated CHB patients. No correlation was observed between the levels of Th1, Th2, and Treg cell-related transcription factors and the HBV DNA copy number (Figure 3).

**Figure 4:**
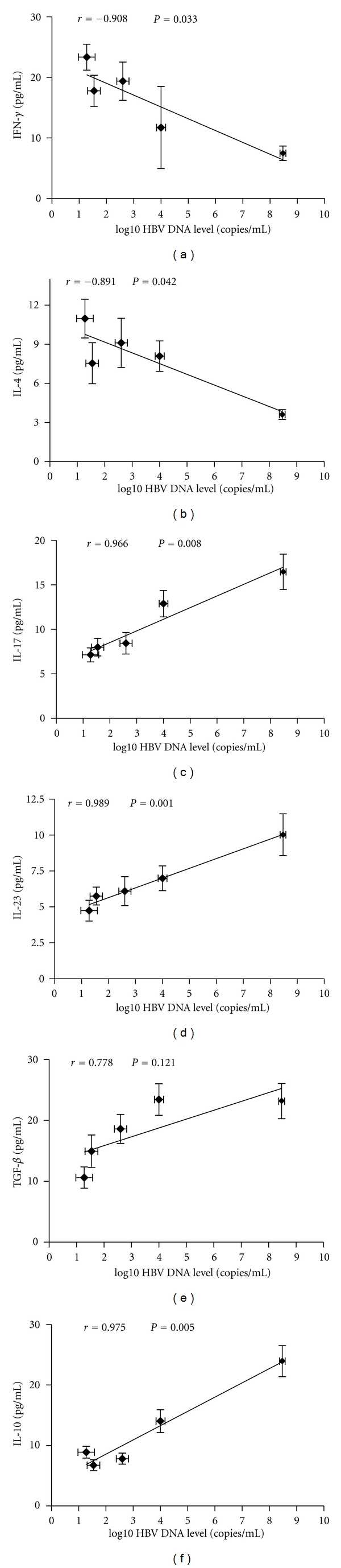
Correlation between the signature cytokines of CD4^+^ T-cell subtypes and HBV DNA level. Expression of the signature cytokines for each of the CD4^+^ T-cell subtypes and HBV DNA level at various time points of treatment (baseline and weeks 12, 24, 36, and 52) are indicated on the plots by diamonds. Error bars indicate SEM.

Accordingly, when the relationship between signature cytokine levels of the CD4^+^ T-cell subtypes and HBV copy number was analyzed, almost no correlation was found for IFN-*γ* or IL-4; however, strong correlations were found for both IL-23 and IL-17 with HBV copy number (Figure 4). For the Treg signature cytokine analysis, no correlation was found between TGF-*β* and HBV copy number, but we did determine that another Treg-related cytokine, IL-10, was correlated significantly with HBV copy number (Figure 4).

## 4. Discussion

Chronic infections eventually led to an exhaustion of the CD8^+^ T-cell population. Evidence has indicated that a robust, early CD4^+^ T-cell response is critical for inducing and sustaining effective CD8^+^ T-cell activity [20, 21]. In HBV infection, CD4^+^ T cells serve as master regulators of the adaptive immune response to HBV [19]. Treatment with the potent nucleoside analogue, lamivudine, leads to reconstitution of CD4^+^ T cell activity and subsequent induction of the CD8^+^ T cell response, as well as decline in the viral load [13, 15]. These observations indicate the key roles played by CD4^+^ T cells in HBV infection.

CD4^+^ T cells are a heterogeneous population, consisting of functionally-distinct Th1, Th2, Treg, and Th17 cells. Thus, we designed the current study to investigate the subpopulation frequencies and related cytokines of each CD4^+^ T subset before and after telbivudine therapy to gain insights into the detailed mechanisms of HBV pathogenesis. The total expression level of cell type-specific transcription factors can reflect the cell frequency directly; thus we measured the T-bet, GATA-3, FOXP3, and ROR*γ*t mRNA levels to represent the frequencies of the four CD4^+^ T-cell subsets, respectively.

Telbivudine is an L-nucleoside that is structurally related to lamivudine and has recently been approved for use in patients with chronic HBV infection. The multinational GLOBE phase III study has shown that telbivudine treatment was superior to lamivudine in its abilities to reduce HBV load to undetectable levels, normalize serum ALT, and improve the rates of HBeAg seroconversion in CHB individuals; moreover, telbivudine was associated with less viral resistance than lamivudine [11, 22, 23]. In the present study, we also observed that telbivudine led to an early rapid viral load reduction in all patients and continued to improve the biochemical and virological parameters throughout the 52 weeks of treatment in patients with CHB, which further provided supporting data for the excellent anti-HBV effects of telbivudine.

It has been reported that Th1 and Th2 immunity are functionally impaired in chronic HBV patients [24], which was also observed at baseline in our current study. We further found that telbivudine-induced suppression of viral replication had little effect on Th1 cells of CHB patients. The Th1 critical transcription factor, T-bet, had a much lower expression level in CHB patients before treatment than in healthy controls, but treatment did not restore the expression to normal levels at any time. However, 52 weeks of treatment led to significant elevation of the Th1 signature cytokine, IFN-*γ*. These results suggested that the observed elevated IFN-*γ* was not principally derived from Th1 cells in these patients, but instead may have originated from other immune cells, such as natural killer T (NKT) cells. It is possible that the antiviral treatment with telbivudine significantly increased the invariant (i) NKT cells, which can produce IFN-*γ* and which are known to have decreased programmed death-1 receptors in virally infected patients [25]. It is also possible that the Th1 cell percentage was actually elevated by telbivudine treatment in our patients, but it is also possible that these cells moved from the periphery to the liver tissue [26], masking detectable changes by our study that relied on PBMC samples. Therefore, it is reasonable that we observed a significant correlation between HBV DNA copy number and IFN-*γ* expressions, but not a marked correlation between HBV DNA copies and T-bet expression during telbivudine treatment.

In contrast to Th1, we found that Th2 cells were correlated with telbivudine-induced suppression of viral replication. The Th2 critical transcription factor, GATA-3, and the Th2 signature cytokine, IL-4, expressions gradually and steadily elevated to reach the normal level after 52 weeks of therapy. Although the increase of GATA-3 expression was not strongly correlated with the decrease of HBV DNA copy number (*P* = 0.055), the IL-4 expression was strongly correlated with HBV DNA copy number (*P* = 0.042). Such relationship was also observed in a previous study of CHB patients during long-term treatment with adefovir dipivoxil [24].

Treg cells play an important role in the impaired immune response of chronic HBV infection. Patients with a chronic HBV infection have increased percentages of CD4^+^CD25^+^ regulatory T cells in their peripheral blood [7, 27]. It has been suggested that Treg can be induced through a repetitive stimulation of T cells by the presence of high concentrations of antigen for longer periods of time [27]. Our data from the current study also showed that the Treg signature transcription factor, FOXP3, expression was higher in CHB patients than in healthy controls. Moreover, telbivudine treatment could rapidly reduce the FOXP3 expression and maintain it extremely low (to almost normal levels) throughout the 52 weeks of treatment, which was similar to the previously reported effect of adefovir on the Treg frequency in CHB patients [28]. We further observed that the expressions of Treg signature cytokines, TGF-*β* and IL-10, were remarkably reduced by telbivudine treatment.

In accordance with our observation, a more recent study also shows a decline in circulating CD4^+^CD25^high^ Tregs together with the decline in viral load and serum ALT normalization after telbivudine treatment [29]. However, that study has not further analyzed the correlation between Tregs frequency and viral load. Here, in this study, statistical analysis did not show any apparent correlation between FOXP3 or TGF-*β* expressions and HBV DNA copy number; instead, there existed significant correlation between the IL-10 level and HBV copy number.

Treg cells are a heterogeneous population and can be subdivided into two specialized types of CD4^+^ Tregs secreting either IL-10 (Tr1) or TGF-*β* (Th3) [30, 31]. In this study, the Tr1 subpopulation was correlated with the HBV copy number, but the Th3 subpopulation was not. It is very possible that the total Treg frequency, which was indicated in this study by total FOXP3 expression, does not correlate with HBV copy number. These results suggest that future analysis should be carried out with each Treg subtype to gain a better understanding of the effects of Treg cells on HBV infection. In addition, the significant decrease of IL-10 that was observed in our study in response to telbivudine treatment was not observed in a previous study of CHB patients treated with adefovir [28], which might reflect the different mechanisms of the two drugs.

Inappropriate, excessive, and nonspecific Th17 effector responses may be involved in the pathogenesis of HBV-associated liver inflammation and hepatocellular damage. Th17 response, especially, may exacerbate the inflammatory processes that lead to liver failure [32]. Our previous study and the observation from Zhang's group showed that IL-17 and IL-23 are highly expressed in CHB patients and are closely related to the serum viral load [33, 34]. In the current study, telbivudine-induced reduction of HBV DNA levels resulted in dramatic decreases of the Th17 critical transcription factor, ROR*γ*t, expression, which was accompanied by a marked decrease in the Th17-related cytokines, IL-17 and IL-23. Statistical analyses indicated that the decrease of Th17 cell frequency and its related cytokines was closely correlated to the reduction of HBV DNA levels, indicating that Th17 cells were the most sensitive subtypes to telbivudine-induced suppression of viral replication and suggesting Th17 cells might represent the most important T cell subtype involved in the pathogenesis of HBV infection.

## 5. Conclusions

The data in this study indicated that telbivudine is able to differentially influence the CD4^+^ T-cell subsets in patients with chronic hepatitis B infection. Such treatment was shown to cause a dramatic decrease in the Th17 and Treg cell frequencies and expressions of their related cytokines. In contrast, treatment caused a dramatic increase in the Th2 cell subtype. While no obvious changes were observed for the Th1 cell frequencies, the subtype-related IFN-*γ* expression was upregulated by telbivudine treatment suggesting an alternative cell source of IFN-*γ* in CHB patients treated with the drug. Statistical analyses indicated that the Th17 and Tr1 cells were the most sensitive subpopulations of the peripheral blood CD4^+^ T cells over the 52 weeks of telbivudine treatment, and might represent suitable and effective markers of responsiveness during telbivudine therapy. Collectively, these findings improve our understanding of hepatitis pathogenesis and may be useful to develop novel therapeutic strategies for controlling viral hepatitis.

##  Authors' Contribution

Y. Zheng, H. Huang and X. Chen contributed equally to this paper. 

## Figures and Tables

**Table 1:** Clinical characteristics of the CHB patients at study enrollment.

Patient characteristics at baseline, *n* = 38	
Sex, male (%)	24 (63)
Age, years^a^	28 (21–50)
HBV DNA, copies/mL^a^	4.6 × 10^8^ (1.4 × 10^7^–4.2 × 10^9^)
ALT^b^, units/L^a^	134.5 (53–549)
HBsAg^a^	13683 (2056–138933)
HBeAg-positive, *n* (%)	38 (100)

ALT: alanine aminotransferase; HBsAg: hepatitis B surface antigen.

^a^Median (range); ^b^normal value: ≤40 IU/L.

**Table 2:** Telbivudine treatment influence on CD4^+^ T-cell subtype-specific cytokines expressions in PBMCs from HBV patients.

Time points (weeks)	IFN-*γ* (pg/mL)	IL-4 (pg/mL)	IL-17 (pg/mL)	IL-23 (pg/mL)	TGF-*β* (pg/mL)	IL-10 (pg/mL)
Baseline	7.459 ± 1.206	3.612 ± 0.370	16.480 ± 1.983	10.030 ± 1.454	23.170 ± 2.891	23.970 ± 2.571
12	11.720 ± 2.566	8.090 ± 1.469**	12.800 ± 1.485	6.990 ± 0.864	23.440 ± 2.593	14.030 ± 1.885**
24	19.380 ± 3.170**	9.100 ± 1.892**	8.440 ± 1.216***	6.100 ± 1.009*	18.610 ± 2.365	7.820 ± 0.908***
36	17.790 ± 2.580**	7.540 ± 1.575*	7.980 ± 0.995***	5.760 ± 0.618**	14.950 ± 2.668*	6.746 ± 0.903***
52	23.350 ± 2.139***	10.900 ± 1.488***	7.140 ± 0.796***	4.740 ± 0.722**	10.610 ± 1.759***	8.890 ± 0.973***
Healthy	26.240 ± 3.066***	14.710 ± 2.080***	5.980 ± 0.605***	3.370 ± 0.517***	8.830 ± 0.931***	3.530 ± 0.332^∗∗∗, #^

Data are shown as mean ± SEM. *P < 0.05, **P < 0.01, and ***P < 0.001 versus   the respective baseline; ^#^ *P* < 0.001 versus 52-week value.
